# Towards validating the hypothesis of phylogenetic profiling

**DOI:** 10.1186/1471-2105-8-S7-S25

**Published:** 2007-11-01

**Authors:** Raja Loganantharaj, Mazen Atwi

**Affiliations:** 1 Bioinformatics Research Lab, The Center for Advanced Computer Studies, PO Box 44330, University of Louisiana, Lafayette, LA 70504, USA

## Abstract

**Background:**

As the number of fully sequenced genome increases, the need is greater for bioinformatics to predict or annotate genes of a newly sequenced genome. Ever since Eisenberg and his colleagues introduced phylogenetic profiling for assigning or predicting protein functions using comparative genomic analysis, the approach has been used in predicting function of some prokaryotic genomes quite successfully. Very little work has been reported in functional prediction of eukaryotes such as mouse and Homo sapiens species from phylogenetic profiles.

**Results:**

We have proposed a general methodology for validating the hypothesis underlying phylogenetic profiling techniques, and have demonstrated it using eukaryotic target genomes such as Homo sapiens and mouse. The gene ontology is used as the *gold standard *for validating functional similarity among the genes in each cluster.

We compute the functional cohesiveness of each cluster and the results appeared to be not encouraging towards finding functionally cohesive phylogenetic profiles. This result complements one recent work on the poor performance on functional linkage in some eukaryotic genome using phylogenetic profiling techniques. If we introduce a broad interpretation for functionally related genes as functional sub-clustering within a phylogenetic profile, then we have a very strong support for the hypothesis as we have shown in the paper.

## Background

The set of genes encoded in a genome plays a pivotal role in the growth, development and survival of a species. Over a long period of evolution, each species has perfected its genome so as to survive and thrive in an adapted environment. A pair of genes are said to be co-evolved if they are consistently present or absent in a set of reference genomes. Such co-evolved genes are believed to share similar functions. Strong functional association among the genes of similar phylogenetic profiles has been reported for some prokaryotic genes such as bacterial organisms [1-3]. Very little work has been done in demonstrating the functional similarity among the genes of a cluster of eukaryotic genomes using phylogenetic profiling technique. In this work we propose a general methodology for empirically validating the hypothesis behind phylogenetic profiling and we have demonstrated the methodology using eukaryotic genomes.

As the numbers of fully sequenced genomes are increasing, the need is greater for bioinformatics to predict or annotate genes of newly sequenced genome. While the final phylogeny of species is not yet completed, it is widely believed that a phylogeny is classified into three domain system namely Archaea, Eukaryota and Bacteria. Large numbers of genomes are fully sequenced by various sequencing centers and many of them are collectively available in few sources such as NCBI and Cogent. For this study, we have downloaded the protein sequences of these completed genomes that include 22 archaea, 24 eukaryotes and 197 Bacteria.

### Basis of phylogenetic profiling

Suppose we are profiling a gene, say *g*, against *n *genomes. A profile of a gene *g *is a binary string of 1 and 0 respectively indicating the presence or the absence of the homolog of *g *in the reference genome corresponding to the position. Let us arrange the k genomes in some order and we denote the genome at position r by genome_r_. If profile_g _[m] denotes the m^th ^value of the profile of gene g then profile_g _[m] = 1 if *g *has a homolog or ortholog in genome_m_, otherwise it is zero.

The profiles of some hypothetical genes of a target genome against some N reference genomes are illustrated in Table 1.

**Table 1:** Example of phylogenetic profiling

Target Genome	Genome_1_	Genome_2_	Genome_3_	.......	Genome_N_
Gene_1_	1	1	0	..	0
Gene_2_	1	0	1	..	1
Gene_k_	0	1	1	..	1

A gene is said to have a homolog in a genome when a gene of the reference genome has a significant alignment score with the target gene. While the alignment can be done with nucleotides or amino acid sequences, we prefer to use amino acid sequences for aligning against the reference genome to avoid further computational complexity due to alternate splicing etc.

### Clustering of genes based on profiling

Several clustering algorithms including K-means, and hierarchical clustering have been used in the literature to group objects with similar patterns. Similarity between a pair of profiles can be measured by Pearson's correlation coefficient or by the cosine angles between the pair of vectors corresponding to the two profiles in a N-dimensional vector space where N is the number of reference genomes, which is also the length of the profile.

The Pearson correlation coefficient between a pair of gene profiles, say *g*_*i *_and *g*_*k*_, is given by

Simij=σij(σi*σj
 MathType@MTEF@5@5@+=feaafiart1ev1aaatCvAUfKttLearuWrP9MDH5MBPbIqV92AaeXatLxBI9gBaebbnrfifHhDYfgasaacH8akY=wiFfYdH8Gipec8Eeeu0xXdbba9frFj0=OqFfea0dXdd9vqai=hGuQ8kuc9pgc9s8qqaq=dirpe0xb9q8qiLsFr0=vr0=vr0dc8meaabaqaciaacaGaaeqabaqabeGadaaakeaacqWGtbWucqWGPbqAcqWGTbqBdaWgaaWcbaGaemyAaKMaemOAaOgabeaakiabg2da9maalaaabaacciGae83Wdm3aaSbaaSqaaiabdMgaPjabdQgaQbqabaaakeaadaGcaaqaaiabcIcaOiab=n8aZnaaBaaaleaacqWGPbqAaeqaaOGaeiOkaOIae83Wdm3aaSbaaSqaaiabdQgaQbqabaaabeaaaaaaaa@41AF@ where *σ*_i,j _is the covariance of the gene profiles of *i *and *j*, and *σ*_i _and *σ*_j _are the standard deviation of the profiles of gene *i *and gene *j *respectively. The similarity has to be computed for each pair of profiles and hence the time complexity of such computation will be O(N^2^).

**Figure 1:**
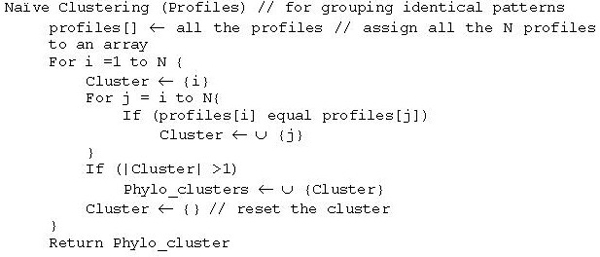
Naïve algorithm for clustering Phylogenetic profiles.

**Figure 2:**
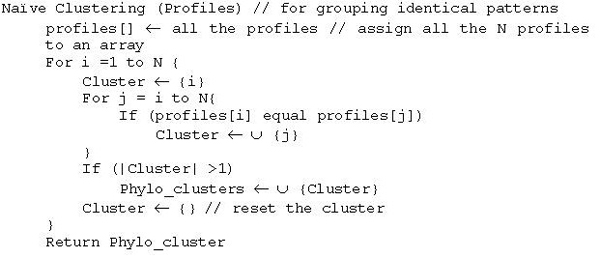
An efficient algorithm for clustering Phylogenetic profiles (O(N Log N)) time.

For the purpose of validating the hypothesis, we group the genes with identical profile patterns and such patterns will have Pearson correlation 1. The complexity of selecting such clusters is O(N) from N by N Pearson correlation matrix. The overall complexity of such clustering algorithm is O(N^2^). We provide pseudo code for such naïve algorithm in Figure 1. The total number of genes in a typical genome is in the order of few thousands, for example, human genome has over 34,000 genes and any improvement in a clustering algorithm will help to reduce the overall time. A careful observation reveals that each pattern encodes an integer and hence identical patterns will map onto the same number. Mapping all the profiles, which are binary strings, onto numbers will take O(N) time and sorting numbers will take O(N log N) time using efficient sorting algorithms such as merge sort. Hence, clustering of profiles based on identical patterns will take O(N log N) time. The pseudo code of an efficient algorithm is given in Figure 2.

### Introduction to gene ontology

**Figure 3:**
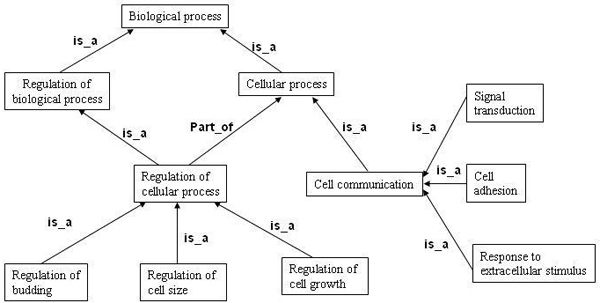
A partial view of gene ontology (GO) terms graph.

The gene ontology (GO) project [4] provides structured controlled vocabularies to address gene products consistently over several databases including FlyBase (*Drosophila*), the *Saccharomyces *Genome Database (SGD) and the Mouse Genome Database (MGD). The ontology describes gene products in terms of their associated biological processes, cellular components and molecular functions for each annotated gene. Each description of a gene product is arranged in a hierarchy from more general to very specific and the corresponding graph forms a directed acyclic graph (DAG) in which each node corresponds to a GO term and the label on the arc corresponds to the relationship between the terms. The relationship between a pair of GO terms includes *part_of *and *is_a*. The DAG in Figure 3 shows a partial view of biological process hierarchy in GO. The terms in any level of the hierarchy inherits all the properties of its ancestors, for example signal transduction is a cellular process.

### Empirical validation

We describe an empirical validation methodology and we demonstrate it by validating the hypothesis underlying phylogenetic profiling: "the co-evolved genes as determined by phylogenetic profiling are functionally related." For validating any hypothesis, a reference or a gold standard must be established. For functional annotations, we use gene annotation, the GO ontology, as a reference or the gold standard.

The work flow of our proposed empirical validation method has the following steps:

1. Select noise free set of sequenced genomes as reference genome

2. Select functionally annotated sequenced genome(s) as target genome(s)

3. Obtain the phylogenetic profile of each gene in the target genome.

4. Cluster the genes based on their profiles

5. In each cluster, remove homolog or group them together so as to form a pseudo gene representing the homolog group

6. Obtain the functional annotation for the genes in each cluster

7. Compute the functional cohesiveness of each cluster

8. If all the clusters are functionally cohesive, the hypothesis is empirically validated.

For each gene from the target genome, a phylogenetic profile against the reference genomes is obtained first. By applying an algorithm described in Figure 2, the profiles are clustered. To avoid homolog genes in a cluster affecting the final results in validating the hypothesis or to test the functional similarity, we group each set of homolog genes of a cluster and consider each group as a single entry in a cluster. Suppose g_11_, g_12_,..., g_1k _are the genes of cluster 1, and g_h1_,.., g_hr _are homolog then the cluster is considered to be consisting of g_11_, g_12_,.., [g_h1_,.., g_hr_],..., g_1k_. This applies for other homolog genes in the cluster.

We test the validity of the hypothesis by testing functional similarity of the genes in a phylogenetic cluster. In terms of GO ontology [5,6], functional similarity refers to both the molecular function and biological processes. Many of the genes in both the mouse and human genome have annotation in gene ontology. Since a gene maps onto many molecular functions, we use average and the best similarity between a pair of genes. Similarly, we apply the same idea to test the biological process similarity.

The annotation of a gene can be obtained through the corresponding GO term(s) associated with the gene. Similarity between a pair of genes becomes the similarity between the sets of GO terms corresponding to the respective genes. GO terms are arranged in a hierarchy from more general to very specific and the corresponding graphs form a directed acyclic graph (DAG). To measure the similarity between a pair of GO terms, one can possibly measure the links between them, but it does not reflect the semantic relations between those terms. We prefer to use information content of nodes with respect to a genome to account for the similarity of pair of GO terms. We start with obtaining the number of times each GO term occurs in a selected target genome. Since GO terms are organized as a directed acyclic graph, a GO term inherits its entire descendant's occurrence. The probability of a GO term, say *t*, occurrence in a genome is obtained by dividing *t*'s occurrence count by the roots occurrence count. The information content of a GO term, *t*, is defined as -log(p_t_) where p_t _is the probability of *t*'s occurrence in the genome.

Using Resnik's [7] approach, similarity between a pair of GO terms is defined as the maximum information content of the subsuming parent and it is defined as

Sim(t1,t2)=max⁡(−log⁡(p(c))c∈S(t1,t2)
 MathType@MTEF@5@5@+=feaafiart1ev1aaatCvAUfKttLearuWrP9MDH5MBPbIqV92AaeXatLxBI9gBaebbnrfifHhDYfgasaacH8akY=wiFfYdH8Gipec8Eeeu0xXdbba9frFj0=OqFfea0dXdd9vqai=hGuQ8kuc9pgc9s8qqaq=dirpe0xb9q8qiLsFr0=vr0=vr0dc8meaabaqaciaacaGaaeqabaqabeGadaaakeaaieaacqWFtbWucqWFPbqAcqWFTbqBcqGGOaakcqWF0baDdaWgaaWcbaGaeGymaedabeaakiabcYcaSiab=rha0naaBaaaleaacqaIYaGmaeqaaOGaeiykaKIaeyypa0ZaaCbeaeaacyGGTbqBcqGGHbqycqGG4baEcqGGOaakcqGHsislcyGGSbaBcqGGVbWBcqGGNbWzcqGGOaakcqWGWbaCcqGGOaakcqWGJbWycqGGPaqkcqGGPaqkaSqaaiabdogaJjabgIGiolabdofatjabcIcaOiabdsha0jabigdaXiabcYcaSiabdsha0jabikdaYiabcMcaPaqabaaaaa@550C@

where S(t_1_, t_2_) is the set of common ancestor terms of *t*_1 _and *t*_2 _in the GO term hierarchy. Using this measure, the similarity varies from 0 to infinity where 0 reflects very little or no similarity.

Lin [8] proposed some variation to Resnik's similarity measure and it normalizes the similarity from 0 to 1. Lin's similarity of pair of terms t_1 _and t_2 _is defined as

Sim(t1,t2)=2×max⁡(log⁡(p(c))c∈S(t1,t2)/(log⁡(p(t1))+log⁡(p(t2)))
 MathType@MTEF@5@5@+=feaafiart1ev1aaatCvAUfKttLearuWrP9MDH5MBPbIqV92AaeXatLxBI9gBaebbnrfifHhDYfgasaacH8akY=wiFfYdH8Gipec8Eeeu0xXdbba9frFj0=OqFfea0dXdd9vqai=hGuQ8kuc9pgc9s8qqaq=dirpe0xb9q8qiLsFr0=vr0=vr0dc8meaabaqaciaacaGaaeqabaqabeGadaaakeaaieaacqWFtbWucqWFPbqAcqWFTbqBcqGGOaakcqWF0baDdaWgaaWcbaGaeGymaedabeaakiabcYcaSiab=rha0naaBaaaleaacqaIYaGmaeqaaOGaeiykaKIaeyypa0JaeGOmaiJaey41aq7aaCbeaeaacyGGTbqBcqGGHbqycqGG4baEcqGGOaakcyGGSbaBcqGGVbWBcqGGNbWzcqGGOaakcqWGWbaCcqGGOaakcqWGJbWycqGGPaqkcqGGPaqkaSqaaiabdogaJjabgIGiolabdofatjabcIcaOiabdsha0jabigdaXiabcYcaSiabdsha0jabikdaYiabcMcaPaqabaGccqGGVaWlcqGGOaakcyGGSbaBcqGGVbWBcqGGNbWzcqGGOaakcqWGWbaCcqGGOaakcqWG0baDdaWgaaWcbaGaeGymaedabeaakiabcMcaPiabcMcaPiabgUcaRiGbcYgaSjabc+gaVjabcEgaNjabcIcaOiabdchaWjabcIcaOiabdsha0naaBaaaleaacqaIYaGmaeqaaOGaeiykaKIaeiykaKIaeiykaKcaaa@71B2@

We use Lin's metric to measure the similarity between a pair of GO terms. A gene usually may have many GO terms, hence to measure the similarity of pair of genes we have to consider all the GO terms corresponding to these pair of genes. Suppose, a gene g_1 _has GO terms t_11_, t_12_,..., t_1r _and g_2 _has GO terms t_21_, t_22_,..., t_2k_. The average similarity of the pair of genes can be defined as the average of all the similarities corresponding to their GO term pairs as defined

Aver_Sim(g1,g2)=∑i=1,j=1i=r,j=kSim(t1i,t2j)/(r×k)
 MathType@MTEF@5@5@+=feaafiart1ev1aaatCvAUfKttLearuWrP9MDH5MBPbIqV92AaeXatLxBI9gBaebbnrfifHhDYfgasaacH8akY=wiFfYdH8Gipec8Eeeu0xXdbba9frFj0=OqFfea0dXdd9vqai=hGuQ8kuc9pgc9s8qqaq=dirpe0xb9q8qiLsFr0=vr0=vr0dc8meaabaqaciaacaGaaeqabaqabeGadaaakeaaieaacqWFbbqqcqWF2bGDcqWFLbqzcqWFYbGCcqWFFbWxcqWFtbWucqWFPbqAcqWFTbqBcqGGOaakcqWFNbWzdaWgaaWcbaGaeGymaedabeaakiabcYcaSiab=DgaNnaaBaaaleaacqaIYaGmaeqaaOGaeiykaKIaeyypa0ZaaabCaeaacqWGtbWucqWGPbqAcqWGTbqBcqGGOaakcqWG0baDdaWgaaWcbaGaeGymaeJaemyAaKgabeaaaeaacqWGPbqAcqGH9aqpcqaIXaqmcqGGSaalcqWGQbGAcqGH9aqpcqaIXaqmaeaacqWGPbqAcqGH9aqpcqWGYbGCcqGGSaalcqWGQbGAcqGH9aqpcqWGRbWAa0GaeyyeIuoakiabcYcaSiabdsha0naaBaaaleaacqaIYaGmcqWGQbGAaeqaaOGaeiykaKIaei4la8IaeiikaGIaemOCaiNaey41aqRaem4AaSMaeiykaKcaaa@67A2@

This represents the average similarity between g_1 _and g_2_. We also can obtain the closest or the best similarity between the pair of genes by taking the best similarity value among the corresponding pairs of GO terms. It is defined as

Best_Sim(g_1_, g_2_) = max_i = 1, r; j = 1, k_(*sim*(*t*_1*i*_, *t*_2*j*_))

Each row of a cluster represents a protein or a group of homolog proteins. We obtain the corresponding GO terms for each row and create average and best similarity matrices for each cluster. Note here that we ignore the proteins that do not have corresponding GO terms. Let Aver_M_k _[*i*, *j*] and Best_M_k _[*i*, *j*] respectively represent average and the best similarity of row *i *and *j *of similarity matrix of cluster *k*. We can compute similarity density for each cluster to measure the cohesiveness of the genes or proteins in a cluster based on their molecular function or biological processes.

Cohesivenessk=2×∑i=1,j=i+1i=n−1,j=nm[i,j]n(n−1)
 MathType@MTEF@5@5@+=feaafiart1ev1aaatCvAUfKttLearuWrP9MDH5MBPbIqV92AaeXatLxBI9gBaebbnrfifHhDYfgasaacH8akY=wiFfYdH8Gipec8Eeeu0xXdbba9frFj0=OqFfea0dXdd9vqai=hGuQ8kuc9pgc9s8qqaq=dirpe0xb9q8qiLsFr0=vr0=vr0dc8meaabaqaciaacaGaaeqabaqabeGadaaakeaaieaacqWFdbWqcqWFVbWBcqWFObaAcqWFLbqzcqWFZbWCcqWFPbqAcqWF2bGDcqWFLbqzcqWFUbGBcqWFLbqzcqWFZbWCcqWFZbWCdaWgaaWcbaGae83AaSgabeaakiabg2da9iabikdaYiabgEna0oaalaaabaWaaabCaeaacqWGTbqBcqGGBbWwcqWGPbqAcqGGSaalcqWGQbGAcqGGDbqxaSqaaiabdMgaPjabg2da9iabigdaXiabcYcaSiabdQgaQjabg2da9iabdMgaPjabgUcaRiabigdaXaqaaiabdMgaPjabg2da9iabd6gaUjabgkHiTiabigdaXiabcYcaSiabdQgaQjabg2da9iabd6gaUbGccqGHris5aaqaaiabd6gaUjabcIcaOiabd6gaUjabgkHiTiabigdaXiabcMcaPaaaaaa@669E@

For average cohesiveness of a cluster, say *k*, *m *[*i*, *j*] represents the average similarity of row *i *and *j *of similarity matrix while for best cohesiveness of the cluster, *m *[*i*, *j*] represents the best similarity of row *i *and *j *ofsimilarity matrix. *n *represents the number of rows of the similarity matrix of cluster *k*.

Cohesiveness of each cluster is obtained so as to validate the hypothesis.

### Objective

The objective of this work is (1) to describe an empirical validation method for testing the hypothesis behind phylogenetic profiling technique with large reference genomes, and (2) to demonstrate the method using eukaryotic target genomes such as mouse and Homo sapiens.

## Results

### Phylogenetic profiles

We have created phylogenetic profiles of mouse and Homo sapiens genomes across the reference genomes using raw sequence score of BLASTP. These profiles with reference to 243 genomes are clustered based on exact profile patterns. To avoid the influence of homolog sequences in a cluster to the final analysis, we group homolog proteins within a cluster and consider each homolog group as a single element or a pseudo gene in a cluster.

### Mapping onto GO terms

In the context of gene ontology a function refers to molecular function and biological process, hence each phylogenetic cluster maps onto the corresponding molecular functional group or biological process group. Each row of a phylogenetic cluster is map onto GO terms (molecular functional or biological process) so as to form molecular functional group or biological process group. We ignored the proteins or rows in a cluster that do not have corresponding GO terms.

### Empirical validation

**Figure 4:**
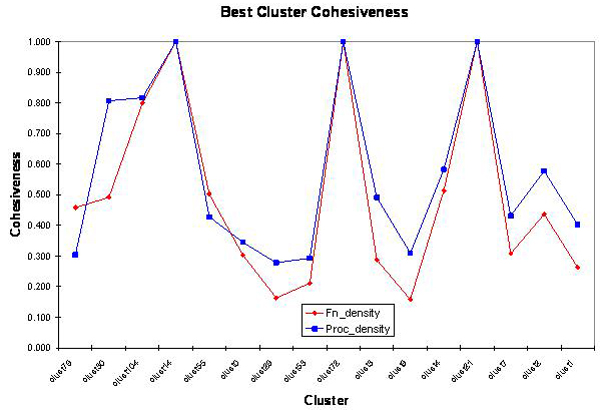
Functional cohesiveness of clusters for Homo sapiens. Table 2 provides the number of genes in each cluster.

We use external or knowledge based validation techniques to test the molecular functional cohesiveness or biological process cohesiveness of each of the phylogenetic clusters. The cohesiveness of a cluster is the same as the similarity density of a cluster. A cluster is said to be functionally cohesive if its cohesiveness or cluster density is closer to 1. Lower value indicates that a cluster has proteins that are functionally diverse. In Table 2, we provide the best and the average cluster cohesiveness of each cluster in Homo sapiens. The functional cohesiveness of clusters for Homo sapiens with the best pairwise similarity is shown in Figure 4.

**Table 2:** Cluster Cohesiveness for homo sapiens genome.

		Cohesiveness with average pairwise similarity		Cohesiveness with the best pairwise similarity	
Cluster	Cluster Elements	Molecular Function	Biological Process	Molecular Function	Biological Process

clust79	4	0.088	0.176	0.458	0.304
clust30	5	0.198	0.318	0.491	0.807
clust104	6	0.309	0.432	0.800	0.816
clust14	6	0.635	0.455	1.000	1.000
clust55	11	0.199	0.168	0.502	0.428
clust0	12	0.139	0.195	0.303	0.345
clust29	15	0.095	0.144	0.163	0.278
clust53	17	0.114	0.174	0.211	0.293
clust72	22	0.625	0.962	1.000	1.000
clust3	24	0.104	0.259	0.287	0.491
clust9	25	0.094	0.194	0.158	0.310
clust4	48	0.154	0.255	0.514	0.583
clust21	64	0.621	0.406	1.000	1.000
clust7	69	0.159	0.226	0.308	0.431
clust2	229	0.145	0.177	0.436	0.578
clust1	396	0.122	0.155	0.262	0.402

A gene usually maps onto many GO terms. To find gene similarity between a pair of genes using GO terms, average pairwise GO terms as well as the best pairwise GO terms are used in the literature. To determine the cluster similarity, it is preferable to use the best pairwise similarity. Among the sixteen clusters, four of them have acceptable close similarity in terms of molecular functions and biological processes. We can state that about 25% of the phylogenetic clusters satisfy functional similarity as per the hypothesis.

**Figure 5:**
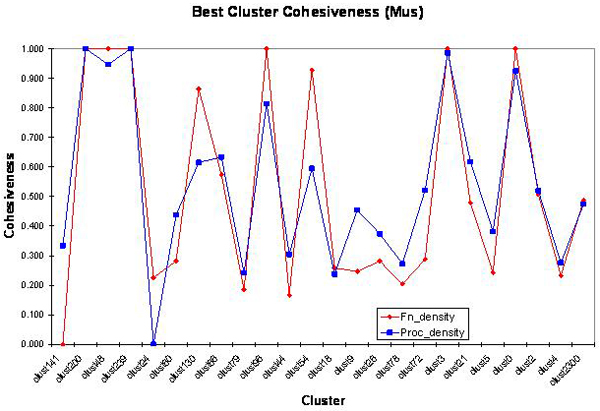
Functional cohesiveness of clusters for Mouse. Table 3 provides the number of genes in each cluster.

In Table 3 we provide the best and the average cluster cohesiveness of each cluster in the mouse genome. The functional cohesiveness of clusters for mouse with the best pairwise similarity is shown in Figure 5.

**Table 3:** Cluster Cohesiveness for mouse genome.

		Cohesiveness with average pairwise similarity		Cohesiveness with the best pairwise similarity	
Cluster	Cluster Elements	Molecular Function	Biological Process	Molecular Function	Biological Process

clust141	3	0.000	0.333	0.000	0.333
clust200	3	0.189	0.280	1.000	1.000
clust48	3	0.326	0.443	1.000	0.946
clust239	4	0.651	0.874	1.000	1.000
clust24	4	0.113	0.000	0.226	0.000
clust60	4	0.208	0.218	0.281	0.438
clust130	5	0.198	0.212	0.864	0.615
clust66	5	0.180	0.172	0.573	0.633
clust79	5	0.072	0.205	0.185	0.241
clust96	5	0.354	0.396	1.000	0.813
clust44	9	0.129	0.146	0.167	0.304
clust54	10	0.215	0.283	0.928	0.595
clust18	11	0.144	0.117	0.259	0.237
clust9	11	0.105	0.205	0.247	0.454
clust26	12	0.091	0.246	0.281	0.373
clust78	15	0.124	0.212	0.204	0.272
clust72	21	0.113	0.295	0.287	0.521
clust3	43	0.908	0.893	1.000	0.986
clust21	59	0.155	0.307	0.479	0.617
clust5	63	0.134	0.211	0.242	0.382
clust0	66	0.751	0.676	1.000	0.925
clust2	205	0.157	0.180	0.507	0.519
clust4	333	0.119	0.142	0.232	0.276
clust2300	434	0.121	0.193	0.487	0.475

Among the twenty four clusters, six of them have acceptable close similarity in terms of molecular functions and biological processes. That is, about 17% of the clusters in the mouse genome satisfy the hypothesis.

**Figure 6:**
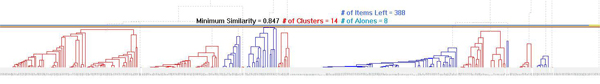
Functional clustering (molecular function) of Clus1 of Home sapiens. Table 4 provides the details on functional cohesiveness.

The cohesiveness of many clusters is quite low and thereby offering weak support to the hypothesis. To understand the functional distribution or associations of the genes within a cluster, we had examined tow largest clusters; clust1 from Homo sapiens and the clust2300 from mouse genome. The genes in each cluster are grouped using functional similarity among the genes as features. We have applied hierarchal clustering [9] and the genes seem to nicely cluster into few functional groups as shown in Figures 6 through 9. The corresponding sub-cluster cohesiveness is shown in Tables 4 and 5.

**Table 4:** Functional cohesiveness of the clusters obtained by using hierarchal clustering method on Phylogenetic Cluster 1 of Homo sapiens.

Molecular Functional clusters from Figure 6			Biological Process Clusters from Figure 7		
Cluster	Cluster Elements	Cohesiveness	Clusters	Cluster Elements	Cohesiveness

clus1f	90	0.538	clust1p	157	0.858
clust3f	50	0.887	clust2p	5	0.642
clust4f	14	0.878	clust3p	46	0.699
clust6f	21	0.508	clust4p	19	0.820
clust7f	8	0.670	clust7p	5	1.000
clust8f	109	0.890	clust8p	80	0.671
clust9f	31	0.907	clust9p	10	0.731
clust11f	15	0.851	clust10p	9	0.972
clust12f	11	0.731	clust11p	33	0.658
clust14f	31	1.000	clust12p	5	1.000
			clust13p	22	1.000

**Table 5:** Functional cohesiveness of the clusters obtained by using hierarchal clustering method on Phylogenetic Cluster 2300 of mouse genome.

Molecular Functional clusters from Figure 8			Biological Process Clusters from Figure 9		
Cluster	Cluster Elements	Cohesiveness	Clusters	Cluster Elements	Cohesiveness

clust1f	139	0.98065	clust1p	202	0.762925
clust2f	71	0.87505	clust2p	11	0.800765
clust3f	6	1	clust3p	55	0.788301
clust4f	27	0.886282	clust5p	44	0.709067
clust5f	7	0.98122	clust7p	28	0.929553
clust6f	7	0.905767	clust9p	59	0.862122
clust7f	8	1	clust10p	11	1
clust8f	64	0.689033			
clust9f	48	0.943796			
clust11f	6	0.707794			
clust12f	26	0.691272			
clust13f	7	1			
clust14f	6	1			

**Figure 7:**
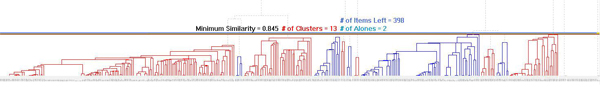
Functional clustering (biological process) of Clus1 of Home sapiens. Table 4 provides the details on functional cohesiveness.

**Figure 8:**
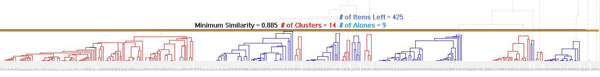
Functional clustering (molecular function) of Clus2300 of mouse genome. Table 5 provides the details on functional cohesiveness.

**Figure 9:**
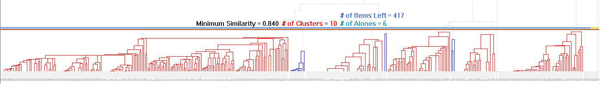
Functional clustering (biological process) of Clus2300 of mouse genome. Table 5 provides the details on functional cohesiveness.

The hierarchal clustering of the 396 genes in the phylogenetic cluster clust1 of Homo sapiens with average link distance and Pearson correlation coefficient is shown in Figures 6 and 7. Even though the overall functional cohesiveness of clust1 is quite low, 0.262 for molecular function and 0.402 for biological process, the functional clustering of clust1 shows remarkable functional cohesiveness as illustrated in Table 4.

The hierarchal clustering of the 434 genes in the phylogenetic cluster clust2300 of mouse genome with average link distance and Perason correlation coefficient is shown in Figures 8 and 9. Even though the overall functional cohesiveness of clust2300 is low, 0.487 for molecular function and 0.475 for biological process, the functional clustering of clust2300 shows remarkable functional cohesiveness as illustrated in Table 5.

## Discussion

Ever since phylogenetic profiling was introduced by Eisenburg's group [1] in 1999, it has been successfully used for predicting functional linkage among genes. Many successful works [2,3,10,11] including the pioneer work was focused on prokaryotes. Very little or no work has been reported in genome wide testing of functional linkage in either homo sapiens or mouse genome using phylogenetic profiling, hence we were unable to compare the current work with others.

The objective of this study is to validate the basic hypothesis of phylogenetic profiling, that is, co-evolved genes as determined by phylogenetic profiling are functionally close together. If the hypothesis would have been true, then genes among a phylogenetic cluster should be functionally close together and thus functional cohesiveness of each cluster must be very high. The results show that phylogenetic profiling does not create functionally cohesive clusters in the mouse or in the Homo sapiens species. The profile is a binary sequence of string of length 243. We use identical profile patterns to cluster the profiles and we have generated over 1,800 clusters in each target genome. When homolog within each cluster was removed or grouped, and clusters with less than three members were filtered out, the phylogenetic clusters reduced to 16 in Homo sapiens and 24 in the mouse genome. In Homo sapiens only 25% of the clusters show close functional cohesiveness while only 17% have similar cohesiveness in the mouse genome. The poor performance can be attributed to many factors including the selection of reference sequences, or the hypothesis may not be valid for a broader class of applications.

Many successful results in predicting functional linkages using phylogenetic profiles were reported on prokaryotic genomes. In those works, very high percentage of reference genomes is also prokaryotes. In the current work, we have created phylogenetic profiles using 243 reference genomes with the following compositions: 22 Archaea, 24 Eukaryotes and 197 Bacteria. Only about 10% of the reference genomes are from Eukaryotes. The lack of eukaryotes in reference genomes may have caused poor results and we cannot verify the statement since there are not many completely sequenced eukaryotes.

Recently Sun et al [2,12] studied composition of reference genome for effective functional prediction in prokaryotic genomes using phylogenetic profiling techniques. We are not sure to what extent the results will be applicable in functional prediction in eukaryotic genomes. We believe it is worth looking into further investigation on selecting reference genome based on some evolutionary history.

Beyond the annotation such as molecular function and biological processes associated with the genes in these clusters, we examined for possible interaction among them. We found only a very few protein to protein interactions in the clusters. It is also possible that these genes in a cluster may be co-regulated or share binding sites. As a future work, we plan to explore the possible regulatory relations among the genes in a cluster.

To understand low cohesiveness in some phylogenetic clusters, we had examined the largest cluster in each target genome. When the genes of the phylogenetic cluster clust1 are functionally clustered using hierarchical method with average linkage and Pearson correlation, they form cohesive functional groups as illustrated in Table 4, and in Figures 6 and 7. Similarly, the genes of the phylogenetic cluster clust2300 form cohesive functional groups as illustrated in Table 5, and in Figures 8 and 9 when clustered using hierarchical method with average linkage and Pearson correlation. This result is very encouraging.

## Conclusion

We have outlined a general methodology for validating the hypothesis behind phylogenetic profiling and have demonstrated it with eukaryotic species mouse and Homo sapiens. We use gene ontology annotations, which has about 60% coverage in the target genomes, for measuring functional density in each phylogenetic cluster.

As we discussed in the previous section, the functional cohesiveness among the clusters are weak in the eukaryotic target genomes, which is in contrast to some spectacular success in functional prediction in prokaryote. While the success in prokaryotic function prediction may be attributed to the large number of prokaryotic reference genome, the failure in eukaryotic function may be attributed to the low number of eukaryotic reference genome, which is only about 10% of the whole reference genome. We anticipate that different mixture of reference sequences based on evolutionary history may help to improve the performance in functional prediction in our target genome. The investigation of transcriptional relations among the clustered genes is underway.

The hypothesis as stated as "co-evolved genes as determined by phylogenetic profiles are functionally related" may be viewed as having two interpretations. The narrow one may state that genes of a phylogenetic cluster must be functionally cohesive and a broad interpretation maybe viewed as the genes of a phylogenetic cluster may belong to few cohesive functional groups. Our results show weak support for the narrow interpretation of the hypothesis while the empirical study shows a strong support for the broad interpretation of the hypothesis. Overall, the phylogenetic profiling is still a very useful technique for predicting function of an unknown protein sequence.

## Methods

### Data sets

We use raw sequence score for obtaining phylogenetic profiles. We have downloaded the protein sequences of 243 fully sequenced genomes consisting of 22 Archaea, 24 Eukaryotes and 197 Bacteria from NCBI and Cogent. We tested the functional links among the clustered genes of the target eukaryotes mouse and the Homo sapiens and verified the underlying hypothesis of phylogenetic profiling.

### Generation of phylogenetic profiles

The phylogenetic profile of a gene can be obtained by determining the presence or the absence of its homolog across different species either by examining orthology databases, such as COG [13,14], or by using raw sequence similarity scores, such as BLASTP [15] E-value. For larger coverage and for automating profile construction, we favored sequence similarity for phylogenetic construction.

We have downloaded BLAST software from the NCBI site [16] and ran BLASTP for each protein sequence in the target genome matching against the sequences from each one of the reference genomes. We use BLOSUM 62 substitution matrix and we set the acceptable E-value threshold to 10^-10^. From each run of BLASTP of a target genome against a reference genome, we construct a column of the profile matrix.

### Clustering of profiles

For each target genome, namely Homo sapiens and mouse we constructed phylogenetic profiles as described in the previous section. Each protein in the target genome is identified or denoted by the sequence GI number and it is represented as the first column in the profile matrix. Each of the remaining 243 columns represents a reference genome.

We cluster profiles of similar patterns together. For the purpose of validating the hypothesis, we use very stringent measurement for clustering, that is, identical patterns are used for clustering. With identical patterns, we gathered large number of clusters over 1,800 in each genome. Some of the genes or the proteins in each cluster may be homolog and they may incorrectly influence the results of validation, hence we remove or group them together by running BLAST on itself for each group of sequences corresponding to each cluster. The substitution matrix and the threshold E-value were set to the same values as used for obtaining phylogenetic profiles. Homologs within a cluster are gathered together and each set forms a row.

### Validation

There are numerous works reported on validating clusters in the literature [17-20]. In this work, we are only interested in validating whether the phylogenetic similarity translates into functional similarity. This is very much similar to external validation or the knowledge directed validation techniques as has been used by the data mining community. The gene ontology (GO) project [4] provides structured controlled vocabularies to address gene products consistently over several databases including our target species. When we talk about functional relations, it precisely translates into either molecular function or biological process or both. For many proteins in our target genome, we can precisely find the corresponding GO term from the GO ontology and hence it provides an elegant way to validate functional similarity among proteins in each cluster.

## Competing interests

The authors declare that they have no competing interests.

## Authors' contributions

Raja Loganantharaj worked on the theory, the procedures and organization behind the work. He also wrote the initial manuscript. Mazen Atwi has set up BLAST and created the profiles. He also implemented GO ontology based similarity system and the WEB interaction interface for it. Both authors read and approved the manuscript.
